# Fluorescent Sensor for PH Monitoring Based on ani-Motif- – Switching Aptamer Containing a Tricyclic Cytosine Analogue (tC)

**DOI:** 10.3390/molecules201018511

**Published:** 2015-10-09

**Authors:** Patrycja Bielecka, Bernard Juskowiak

**Affiliations:** Laboratory of Bioanalytical Chemistry, Faculty of Chemistry, Adam Mickiewicz University, Umultowska 89b 61-614 Poznan, Poland; E-Mail: rzepeckap@gmail.com

**Keywords:** aptamer probe, fluorescent cytosine analogue, i-motif, pH sensing

## Abstract

There are cytosine-rich regions in the genome that bind protons with high specificity. Thus protonated C-rich sequence may undergo folding to tetraplex structures called i-motifs. Therefore, one can regard such specific C-rich oligonucleotides as aptamers that recognize protons and undergo conformational transitions. Proper labeling of the aptamer with a fluorescent tag constitutes a platform to construct a pH-sensitive aptasensor. Since the hemiprotonated C-C^+^ base pairs are responsible for the folded tetraplex structure of i-motif, we decided to substitute one of cytosines in an aptamer sequence with its fluorescent analogue, 1,3-diaza-2-oxophenothiazine (tC). In this paper we report on three tC-modified fluorescent probes that contain RET related sequences as a proton recognizing aptamer. Results of the circular dichroism (CD), UV absorption melting experiments, and steady-state fluorescence measurements of these tC-modified i-motif probes are presented and discussed. The pH-induced i-motif formation by the probes resulted in fluorescence quenching of tC fluorophore. Efficiency of quenching was related to the pH variations. Suitability of the sensor for monitoring pH changes was also demonstrated.

## 1. Introduction

Aptamers are single-stranded oligonucleotide (DNA or RNA) probes that are able to specifically bind target analytes with high affinities. They have recently been exploited as recognition elements for the development of biosensors and bioassays [1]. Aptamers can be generated by an *in vitro* procedure called SELEX [2] to bind diverse targets including metal ions, simple organic molecules, proteins or even whole live cells. Interestingly, there are in genome cytosine-rich sequences that bind protons with high specificity. Moreover, thus protonated C-rich sequence undergoes folding to form three dimensional tetraplex structures called i-motifs. Slightly acidic or even neutral pH is sufficient to induce i-motif formation. Therefore, one can regard these specific C-rich oligonucleotides as aptamers that recognize protons and undergo conformational transitions. Formation of i-motif structures was first observed for the d-TCCCCC oligonucleotide [3,4]. Depending on the number of cytosines in a strand, an inter- or intramolecular i-tetraplex may evolve [3,4,5,6,7]. The i-motif structures have been reported to form for telomeric DNA sequences [5,6,7] and in the promoter regions of different cancer-related genes, such as c-myc [8], Rb [9], RET [10], VEGF [11], and Bcl-2 [12]. The typical examples of i-motif structures are predominant in the acidic pH region, with the transition midpoint at about pH 6.5, but it often depends on the sequence and environmental conditions [13,14]. It is expected that the higher the pH above the pK_a_ of cytosine (pK_a_ = 4.3 for free cytosine [15]), the more difficult the formation of the i-motif. It is well known that temperature, pH and solvent conditions have an impact on the stability of i-motifs [16,17]. Moreover, the i-tetraplex stability is clearly related to the sequence composition, *i.e.*, the number of cytosines in repeating C-tracts or length of loops [18,19]. It has been reported that cytosine-rich sequences can form i-motif structures at neutral or even slightly alkaline pH, but only at low temperature [20] or when molecular crowding agents are present [14,21]. Because of these very interesting properties, i-motif structures have great potential applications in nanotechnology as molecular switches, biosensors, and nanomachines [22].

The visualization of conformational transitions in aptamer-based sensors with fluorescence labeling has been extensively used [1,22,23,24,25,26]. Most of these labeling strategies are based on the attachment of fluorescent tags and exploit fluorescence enhancement, quenching, anisotropy change, FRET signal or excimer emission approaches [23,25,26]. Substitution of normal nucleobases with their fluorescent analogues is not a popular approach because of the limited number of available fluorescent nucleobases [27,28,29,30]. Fortunately, cytosine that is the main nucleobase in i-motif-forming oligonucleotides can be substituted with tricyclic derivatives of phenothiazine or phenoxazine: tC (1,3-diaza-2-oxophenothiazine), tC° (1,3-diaza-2-oxophenoxazine) or tC_nitro_ (7-nitro-1,3-diaza-2-oxophenothiazine). We decided to modify our aptamer for pH sensing with a tC analogue, as this nucleobase has not been tested with i-motif structures yet. The fluorescence quantum yield of tC is relatively unchanged when incorporated in single- and double-stranded DNA, irrespective of the neighboring bases [29,30]. The nitro-substituted, tC_nitro_ is virtually non-fluorescent in polar solvents at room temperature [31]. The red-shifted absorption of tC_nitro_ has a considerable spectral overlap with the fluorescence spectra of both tC and tC°, thus it can be used as a FRET partner with tC or tC° in nucleic acid studies [32]. Additionally, these synthetic analogues are very similar in both size and shape to cytosine and their incorporation into single- or double-stranded DNA induces minimal perturbations to the overall structure [33]. Moreover, it was shown that pH, salt, and temperature have very limited effects on their photophysics under biological conditions [31]. The incorporation of tC analogue into DNA oligomers gives a higher or unchanged *T*_m_ (overage increase ~2.7 °C), so it promotes a stabilization of the structures [33]. Recently, Reilly and co-workers showed that a fluorescent 1,3-diaza-2-oxophenoxazine (tC°) analogue could be used to monitor conformational transition in the i-motif structure of a 20-mer mutant of c-myc strand [34].

This paper describes results of characterization of new pH aptamer probes able to form i-motif structures. They are based on a C-rich aptamer related to the RET sequence: C_4_GC_4_GC_4_GC_4_A (RET20) substituted with the cytosine fluorescent analogue tC at three different sites: at the 1st, 3rd and 6th positions from the 5′ end. The RET20 sequence was chosen taking into account three factors: (i) documented uniform folding of the i-motif structure [10], (ii) presence of cytosines with potentially different structural roles (outside i-motif, involved in base-pairing and in a loop), which could be substituted with tC, (iii) high thermal stability of the unmodified RET20 sequence. A reference sequence (majority of thymine bases) is also studied (Table 1). Comparative studies at several pH (5.5–8.0) and different temperatures (15–90 °C) were carried out using UV-Vis, circular dichroism (CD), and fluorescence spectroscopies. For each probe melting temperatures at different pH were determined and pH value of transition between i-motif and a random coil structures (pH_T_) were assessed. Performance of the probes as pH fluorescent sensors was examined by determining linear response range and reversibility of fluorescence readout to pH cycling.

## 2. Results and Discussion

### 2.1. Design of tC-Modified i-Motifs

The modified oligonucleotides used in this study are collected in Table 1. All cytosine-rich sequences contained a 20-mer domain found in RET proto-oncogene that was proved to self-assemble into intramolecular i-motif in acidic pH [10]. A reference probe TtC contains mostly thymine nucleobases and was design for comparison purposes since TtC probe is unable to form i-motif structure.

**Table 1:** Oligonucleotide sequences and their modification with tC.

Name	Sequence 5′-3′	Position of tC Analogue
1tC	tCCC CGC CCC GCC CCG CCC CA	1
3tC	CCtC CGC CCC GCC CCG CCC CA	3
6tC	CCC CGtC CCC GCC CCG CCC CA	6
TtC	tCCC TTT TTT TTT TTT TTT TT	1

**Figure 1:**
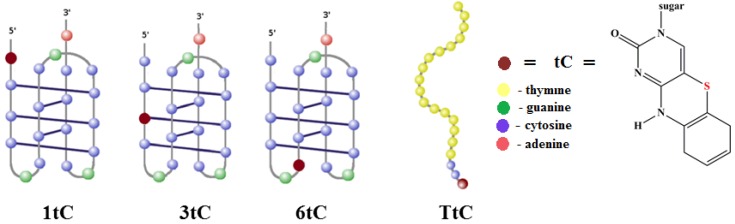
Structures of intramolecular i-motifs formed by 1tC, 3tC, and 6tC probes and single stranded structure of TtC probe. The positions of the fluorescent cytosine analogue are marked with brown circles, cytosine is depicted as blue circle, thymine—as yellow circle, guanine—as green circle and adenine—as red circle.

Different positions of tC substitution were designed to test the effect of incorporation of tC on the thermal stability and spectral properties of these fluorescent probes. As illustrated in Figure 1, the substitution of tC at the 1st position (1tC) locates the fluorescent tag outside the i-motif structure. Also internal substitution in a loop at the 6th position (6tC) protects tC from base pairing with cytosine. In contrast, internal substitution at the 3rd position (3tC probe) should force the tC nucleobase to participate in i-motif stabilization through base pairing with the cytosine at the 13th position in the i-motif core (Figure 1).

The working principle of tC-labeled i-motif probes is based on a switching process between an extended form of the probe dominating at higher pH and a compact i-motif structure adopted at lower pH values. Fluorescence quantum yield of tC is nearly unaffected by the environmental conditions (salt content, stacking or neighborhood of other nucleobases [31]), and the only process that seriously quench tC fluorescence is protonation of N3 nitrogen [28,31]. Formation of i-motif due to partial protonation of cytosines is expected to alter also protonation equilibrium of tC, which should quench tC fluorescence with efficiency dependent of the pH value of the environment. The proposed i-motif-based sensors should give an analytical response to pH lowering.

### 2.2. Thermal Stability of i-Motifs

The denaturation experiments were performed at different pH values in order to evaluate the thermal stability of the i-motif probes and to assess the effect of tC substitution position. Ultraviolet (UV) absorption changes at different temperatures provide information on the kinetics and on the thermodynamics of formation and unfolding of tetraplex structures.

**Figure 2:**
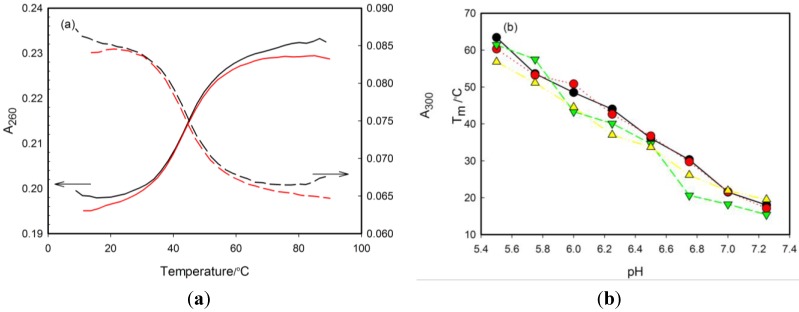
(**a**) The melting profile of 1tC probe at the pH 6.25. The presented curves illustrate cooling (black lines) and heating (red lines) experiments recorded at 260 nm (solid lines) and at 300 nm (dashes lines). The scales for the 260 nm and 300 nm readings are notably different. (**b**) Temperature melting values of tC-modified– probes: 1tC (red), 3tC (green) and 6tC (yellow) and unmodified 5′-(C_4_G)_3_C_4_A-3′ sequence (black) plotted against pH.

The thermal denaturation plots of all probes exhibited hyperchromism at 260 nm and hypochromism at 300 nm (Figure 2a) that is consistent with data of Mergny *et al.* concerning formation of i-motif structures (respective wavelengths, 265 and 295 nm) [6]. The analysis of the 260 and 300 nm absorbance profiles led to identical melting temperatures (*T*_m_). Melting experiments indicated that *T*_m_ values determined for all studied oligonucleotides were definitely higher in the slightly acidic pH range. Hence, at pH values from 5.50 to 7.25 the stability of the i-motif structure is a linear function of pH and the *T*_m_ decreases when pH increases (Table 2) and Figure 2b). At pH 7.50, 7.75 and 8.00, the probes appeared to form lower-stability folded structures with *T*_m_ below 15 °C.

The unmodified RET20 oligonucleotide behaves very similar to the three tC-substituted analogues showing a *T*_m_ increase from 18.0 °C to 63.4 °C with pH drop from 7.25 to 5.50. Moreover, we observed only small differences in melting temperatures between tC probes and unlabeled strand at all pH values: for 1tC probe *T*_m_ increased from 17.1 °C to 60.3 °C; for 3tC probe, from 15.4 °C to 61.4 °C; and for 6tC probe, from 19.6 °C to 59.1 °C in the same pH range. The assessment of stabilizing/destabilizing effect of tC incorporation into RET20 oligonucleotide is difficult when one considers small negative/positive Δ*T*_m_ differences for particular pH values and limited precision of *T*_m_ measurements (mean values of four determinations with RSD of *ca*. 3%). Therefore, one can conclude that incorporation of tC outside of the i-motif core (1tC and 6tC probes) exerted negligible effect on the stability of i-motif structure. On the other hand, careful inspection of results for 3tC probe (Table 2) revealed that involvement of tC in the hydrogen-bonding interactions destabilized slightly i-motif structure, especially in the higher pH values (pH 6.0–7.25) as for this probe the greatest difference in melting temperatures comparing to unmodified strands were observed. For example, *T*_m_ of 3tC probe is 5.2 °C and 9.4 °C degrees lower than in case of RET20 sequence in pH 6.00 and 6.75, respectively.

### 2.3. Effect of pH on CD Spectra

**Figure 3:**
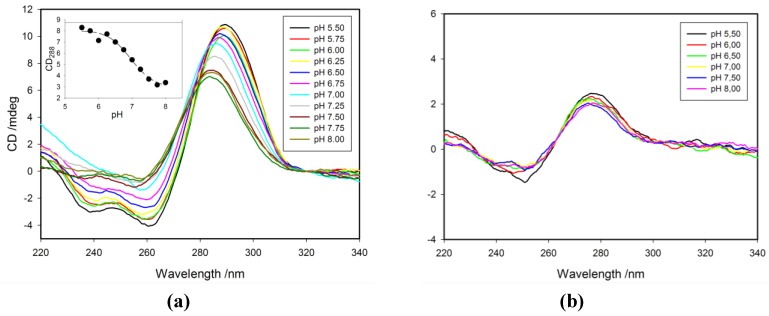
(**a**) CD spectra of 1tC probe in the pH range of 5.50–8.00. Insert: the dependence of CD signal at 288 nm against the pH values. The pH_T_ values for this and other probes are shown in Table 2. (**b**) CD spectra of TtC probe at different pH.

We recorded circular dichroism (CD) spectra to follow independently the conformational transition between the i-motif and the random coil single stranded structure (ssDNA). The CD spectra of 1tC probe in acidic solution show a positive signal at 288 nm and a negative signal at 260 nm, indicative of a folded i-motif structure [10,26,35] (Figure 3a). Similar spectral signatures were observed for other probes (data not shown). When this i-motif structure undergoes unfolding at higher pH values (pH > 7.00), the intensity of positive peak decreases with a blue shift, and simultaneously the negative peak disappears. The spectra recorded at pH values close to 8.0 exhibit a weak positive band around 284 nm that is typical for random coil single-stranded DNA. Spectra shown in Figure 3b, measured for the TtC probe, lacks characteristic signals for the i-motif structure in accordance with the absence of i-motif for the reference sequence.

**Table 2:** Determined values of *T*_m_, Δ*T*_m_,_R20-XtC_ and pH_T_ for all probes at pH range 5.5–8.0 (R20 denotes RET20 sequence, X = 1, 3 or 6 position).

	pH 5.50	pH 5.75	pH 6.00	pH 6.25	pH 6.50	pH 6.75	pH 7.00	pH 7.25	pH 7.50	pH 7.75	pH 8.00	pH_T_
RET20	63.4 ± 1.5	53.6 ± 1.3	48.5 ± 1.5	44.0 ± 1.4	36.1 ± 1.2	30.3 ± 1.1	21.5 ± 1.2	18.0 ± 0.8	<15	<15	<15	7.1 ± 0.2
1tC	60.3 ± 1.8	53.2 ± 1.5	50.8 ± 1.4	42.6 ± 1.1	36.7 ± 1.0	29.7 ± 0.8	21.6 ± 0.8	17.1 ± 0.7	<15	<15	<15	6.9 ± 0.2
ΔT_m_,_R20-1tC_	3.1	0.4	−2.3	1.4	−0.6	0.6	−0.1	0.9	-	-	-	-
3tC	61.4 ± 1.5	57.5 ± 1.9	43.3 ± 1.3	40.1 ± 1.4	34.6 ± 1.5	20.6 ± 1.2	18.2 ± 0.7	15.4 ± 0.8	<15	<15	<15	6.9 ± 0.2
ΔT_m_,_R20-3tC_	2.0	−3.9	5.2	3.9	1.5	9.4	3.3	2.6	-	-	-	-
6tC	59.1 ± 1.9	57.3 ± 1.7	47.0 ± 1.2	42.9 ± 1.1	36.1 ± 1.1	26.7 ± 1.4	21.3 ± 0.6	19.6 ± 0.7	<15	<15	<15	7.1 ± 0.2
ΔT_m_,_R20-6tC_	4.3	−3.7	1.5	1.1	0.0	3.6	0.2	−1.6	-	-	-	-

The widely used parameter that characterizes the ability of a cytosine-rich strand to fold into i-motif is the pKa value of the i-motif. It is generally determined from a CD signal plotted against pH and fitting to a sigmoidal function for a two-state model, folded i-motif and unfolded random coil structure. Thus the determined pKa value is a conditional constant that actually does not represent a dissociation process written according to commonly accepted scheme: HnL←→ nH + L. This parameter should be rather regarded as a pH of conformational transition (pH_T_). At the pH equal to pH_T_, 50% of probe is in a protonated folded i-motif state and 50% remains as a nonprotonated random coil. Considering that only half of the cytosines in a strand that fold into an i-motif are protonated (hemiprotonated C-C^+^ base-pairs), one can calculate that *ca*. 25% of cytosine content in a sample solution is protonated at pH = pH_T_. Therefore, the use of pK_a_ to characterize conformational transition of C-rich oligos is confusing and we decided instead to use the term pH_T_ to describe the pH-transition midpoint of the i-motif. To determine pH_T_ values for our probes, the CD signal observed at 288 nm was plotted against pH and fitted into a sigmoidal function. The obtained pH_T_ values are given in Table 2 and representative graph is shown in Figure 3a (insert). Sigmoidal type curves were obtained at 288 nm for all probes with a midpoint near the pH of 7.00. For example, 6tC and RET20 (unmodified) possess pH_T_ values of 7.1, while 1tC and 3tC have slightly lower pH_T_ of 6.9. Taking into account the experimental error of pH_T_ determination (±0.2), these data suggest that the substitution of cytosine for fluorescent analogue has a negligible effect on the pH_T_ and this conclusion is consistent with the low dependence of *T*_m_ values of the substitution position of tC. Reilly *et al.* [34] reported similar observation for the c-myc sequences substituted with a fluorescent tC° analogue.

### 2.4. Fluorescence Properties of tC Modified Probes

**Figure 4:**
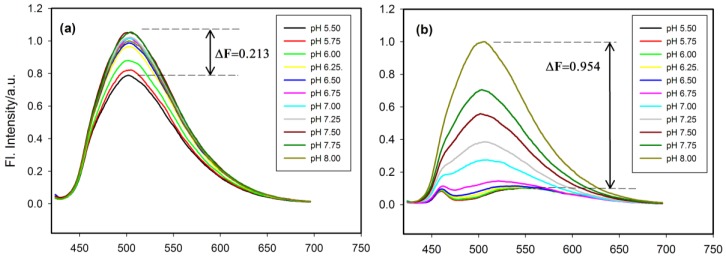

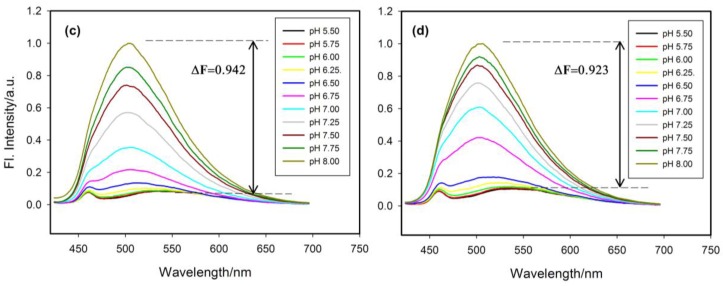
The emission spectra of tC-based probes recorded at room temperature at the pH range from 5.50 to 8.00 with λ_ex_ = 395 nm: (**a**) TtC probe, (**b**) 1tC probe, (**c**) 3tC probe and (**d**) 6tC probe. The ΔF values were calculated as F_pH8.0_–F_pH5.5_.

Fluorescence spectra for tC-modified probes were recorded at multiple pH values and results are shown in Figure 4. The tC fluorophore was excited at its maximum of absorption (395 nm). In all cases probes showed fluorescence band with pronounced intensity (λ_max_ = 505 nm) at alkaline or neutral pH values. The emission band underwent gradual quenching upon lowering pH from 8.0 to 5.5.

**Figure 5:**
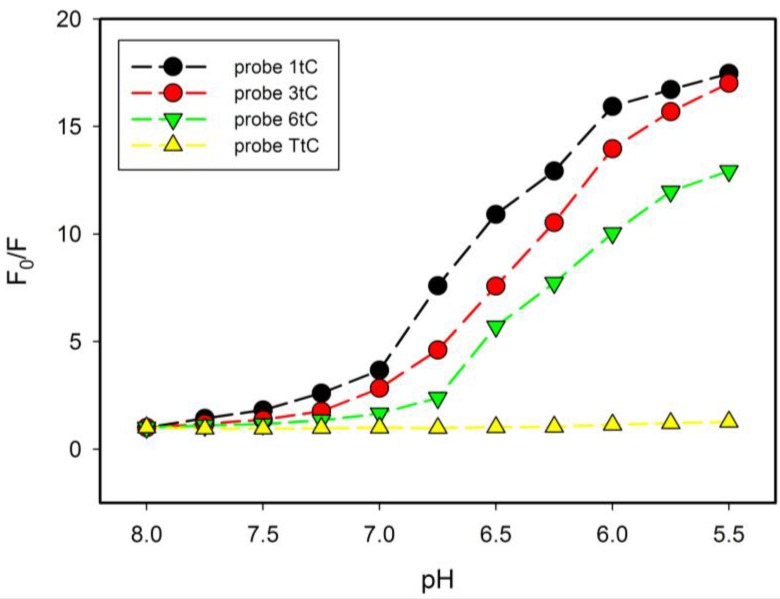
The dependence of fluorescence quenching (F_0_/F) *vs.* pH for 1tC (black circles), 3tC (red circles), 6tC probes (green triangles down) and TtC (yellow triangles up). F_0_ (at 505 nm) is the fluorescence of tC probe at pH 8.00 and F is the fluorescence intensity of tC probe at a particular pH value.

The observed insensitivity of the fluorescence λ_max_ to the position of substitution is in good agreement with results reported for miscellaneous tC-modified single-stranded DNA and duplexes [30,36]. This emission band is characteristic for the neutral form of tC fluorophore [28]. Interestingly, a substantial quenching and red shift in the emission band is observed for acidic solution of i-motif probes (Figure 4b–d) with λ_max_ shifted to *ca*. 540 nm. This efficiently quenched fluorescence should be ascribed to the protonated tC-H^+^ fluorophore since similar quenching and red shift were reported for tC nucleoside in strongly acidic solution (pH = 0.5) [28]. It should be noted, that reference TtC oligo at the same low pH value (5.5) exhibited intense fluorescence without any red shift, which suggested that neutral form of tC was present in the case of probe with random coil conformation (Figure 4a). All these results indicate that i-motif assembly plays crucial role in protonation and efficient quenching of tC fluorescence in the 1tC, 3tC, and 6tC probes. Considering very low pK_a_ of tC nucleoside (pK_a_ ~1 [28]), one can anticipate that intrinsic concentration of protons inside the i-motif core should be at least 4–5 orders lower than pH in bulk solution (pH 5.5–6.0). This is plausible conclusion if one considers that five hemiprotonated C-C^+^ base pairs stabilize i-motif of RET 20 oligonucleotide [10]. Unlike the position of tC emission band, fluorescence intensity was seriously affected by pH variation. As shown in Figure 4, three of the tC substituted probes (1tC, 3tC and 6tC) exhibited very similar ΔF values of 0.944, 0.942, and 0.923, respectively. In the case of TtC probe, a decrease in emission was modest, ΔF approached 0.213 upon the pH change from 8.00 to 5.50 (Figure 4a). These results indicate that the amplitude of fluorescence intensity changes with pH for i-motif-based probes is somehow dependent of the position of substitution (1tC *vs.* 3tC *vs.* 6tC). In order to clearly identify these differences, we plotted a fluorescence ratio defined as F_0_/F (where F_0_ denotes fluorescence intensity at pH 8.00 and F–that at particular pH) and results are shown in Figure 5.

One cannot see any noticeable changes for the TtC probe in the studied pH range of 8.0–5.5 (yellow triangles in Figure 5). As expected, the i-motif based probes (1tC, 3tC, 6tC) exhibited a dramatic increase in efficiency of quenching at pH values below 7.0. The most significant changes can be observed for 1tC probe possessing tC analogue substituted at the 5′ end (not involved in base pairing with cytosine). The order of pH dependent quenching for tC probes is as follows: 6tC < 3tC < 1tC. This order is inconsistent with the values of pH_T_ determined from CD data. It means that not only protonation of tC nucleobase but also other processes are involved in quenching. Interestingly, highest quenching efficiency was observed for 5′-end substituted 1tC probe, which is expected to possess the highest rotational freedom.

**Figure 6:**
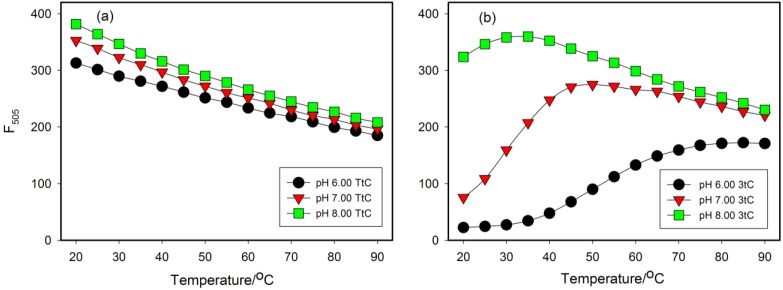
Temperature effect on the fluorescence intensity for TtC probe (**a**) and 3tC probe (**b**) at pH 6.00 (circles), 7.00 (triangles) and 8.00 (squares).

Additionally, we have recorded dependence of fluorescence intensity *versus* temperature for all probes at pH 6.00, 7.00 and 8.00. Representative results for probes 3tC and TtC are shown in Figure 6. Temperature profiles for TtC probe are similar at all pH values and show gradual fluorescence quenching with an increase in temperature (Figure 6a). Such dependence is typical for fluorophores, for which an increase in temperature causes enhancement of nonradiative excited state deactivation processes e.g., internal conversion. Decrease in tC nucleoside fluorescence quantum yield with temperature has been reported in MeTHF glass [30]. In contrast, temperature dependent fluorescence profiles for i-motif-based probes were more complex and depended on pH as illustrated on the example of for 3tC probe (Figure 6b). At pH 6.0 the probe exists mainly in an i-motif form with totally quenched fluorescence at low temperature. An increase in temperature caused unfolding of i-motif, thus dequenching plot that resembles melting profile is observed. At pH 7.0 the probe is only partially in a folded form and only the folded fraction undergoes melting (fluorescence increases with temperature rise up to about 50 °C). Further increase in temperature exerts similar effect as that observed for random coil TtC. Finally, at pH 8.0 only small fraction of probe is in a folded form, thus the two competing processes (*i.e.*, dequenching associated with unfolding and temperature induced non-radiative decay) produce fluorescence plot with a maximum shifted to about 30 °C. Concluding, the interplay of many processes (i-motif folding, tC protonation, nucleobases stacking, thermal deactivation of tC excited states) may affect tC fluorescence, therefore temperature profiles of fluorescence cannot be directly exploited for melting temperature determination.

**Figure 7:**
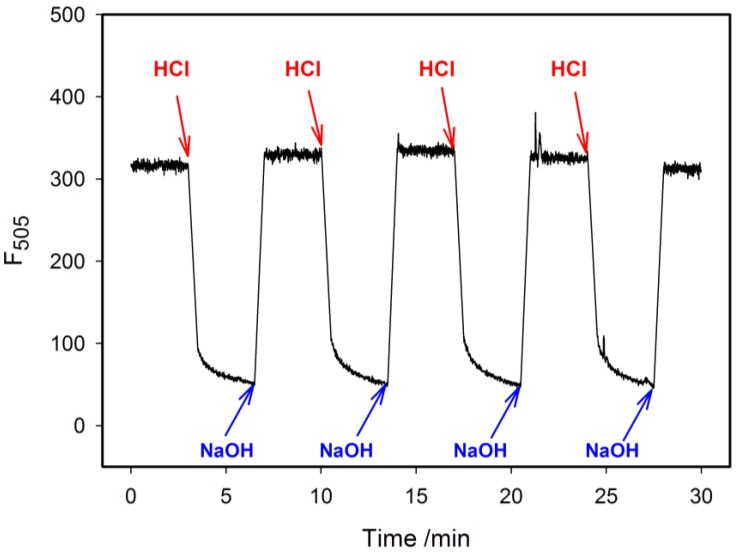
Reversible fluoresce response of 6tC probe to cyclic pH changes between 7.5 and 5.5. Fluorescence intensity was measured at 505 nm with λ_ex_ = 395 nm at room temperature.

Since the main purpose of our study was to develop and characterize a fluorescent i-motif probe that could be potentially suitable for cellular applications, we attempted to test whether the probes with incorporated tC cytosine analogue were capable of monitoring reversibly pH changes induced by alternate addition of acid and base solutions. The experiment was performed with pH cycling between 7.5 ± 0.3 and 5.5 ± 0.3 by addition small amounts of 1M HCl or 1M NaOH five times (Figure 7). Upon such pH changes the probe is supposed to undergo conformational switching between an “open” single-stranded form (pH 7.5) and a “closed” i-motif structure (pH 5.5). As expected, we observed an increase in fluorescence after pH rise that corresponded to transition between closed and open state of probe and a drop in fluorescence upon pH decrease. It should be noted that the fluorescence changes were reversible and that i-motif folding (lower pH) needed a longer equilibration time comparing to unfolding process (higher pH). According to kinetic data of i-motif formation [20,37], both folding and unfolding processes should be rather fast at the pH values used in this experiment. Hence, the slow fluorescence changes observed upon pH lowering did not represent i-motif folding kinetics but rather the process of tC protonation. What is important, the pH changes can be monitored in the range relevant to biological systems. For example, the probe 1tC exhibits linear response of fluorescence within pH 6.0 and 7.0 with high resolution below 0.1 pH.

## 3. Experimental Section 

### 3.1. Oligonucleotides

The DNA oligonucleotides d(C_4_G)_3_C_4_A modified with fluorescent cytosine analogue tC (Table 1) were synthesized and HPLC purified by Future Synthesis, Poznan, Poland. The strand concentration was determined from the absorbance measured at neutral pH and elevated temperature of 85 °C using the ε_260_ values of 164,900 M^−1^ cm^−1^ (for 1tC, 3tCand 6tC) and 166700 M^−1^ cm^−1^ (for TtC). All experiments were performed in a 10 mM sodium cacodylate buffer with pH ranging from 5.50 to 8.00. Before spectral measurements, the solution of oligonucleotides in appropriate buffer solutions were annealed by being heated at 90 °C for 5 min and then slowly cooled to room temperature.

### 3.2. UV Melting Experiments

Thermal melting curves were recorded on a Cary 100 spectrophotometer (Agilent Technologies, Mulgrave, VIC, Australia) equipped with a Peltier temperature control accessory. Absorbance *versus* temperature plots (heating and cooling runs) were measured at 300 nm and 260 nm. The temperature was programmed to change reversibly between 10 and 90 °C with a rate of 1 °C/min. The melting temperatures (*T*_m_) were determined as the maximum of the first derivative of the melting curves. Each *T*_m_ value was an average of four independent measurements.

### 3.3. Circular Dichroism (CD)

Circular dichroism spectra were recorded at 25 °C on a Jasco J-810 spectropolarimeter equipped with a Peltier accessory (Jasco, Kyoto, Japan). Each spectrum represented an average of three scans accumulated with a scan speed of 200 nm/min from 220 to 360 nm. The corresponding cacodylate buffer (10 mM) was used as a blank solution. A spectrum of buffer solution was subtracted from the spectra of investigated probes. CD spectra were recorded at different pH (5.5–8.0) at strand concentration of 2 μM. The spectra were smoothed using manufacturer software.

### 3.4. Fluorescence Measurements

Fluorescence measurements were carried out on a spectrofluorometer model Cary Eclipse (Agilent Technologies, Mulgrave, VIC, Australia) in the spectral range of 420–700 nm in a 10 mm quartz cell (2 mL sample solution). Each probe was diluted with a 10 mM cacodylate buffer to a strand concentration of 1.0 µM. All fluorescence measurements were performed under the same spectral conditions: the slit width for excitation was 10 nm and that for emission was 5 nm, and the excitation wavelength was set at 395 nm. Temperature-dependent emission spectra were recorded every 5 °C from 20 °C to 90 °C. The pH cycling experiments were performed for 30 min at room temperature. The sample pH was cycled between pH 5.5 and 7.5 by alternate addition of 8 μL 1.0 M HCl and 8 μL of 1.0 M NaOH, respectively.

## 4. Conclusions 

We have designed and characterized three new pH-sensitive aptamer probes able to form i-motif structures. They are based on a C-rich aptamer related to the RET sequence: C_4_GC_4_GC_4_GC_4_A substituted with the cytosine fluorescent analogue 1,3-diaza-2-oxophenothiazine (tC) substituted at three different sites: at the 1st, 3rd and 6th position from the 5′ end. Melting temperatures determined at several pH values indicated that thermal stability of the probes was not seriously affected by the tC analogue incorporation and did not depend on the substitution position, except for 3tC probe that showed slightly destabilized i-motif structure, especially at the higher pH values (pH 6.0–7.25). CD spectra confirmed the expected working principle of the probes, which consisted in a switching process between an extended form of the probe dominating at higher pH and a compact i-motif structure adopted at lower pH values. The conformational transition midpoint (pH_T_) did not vary with tC on the substitution and its position. Probes showed fluorescence band with pronounced intensity (λ_max_ = 505 nm) at alkaline or neutral pH values. The emission band underwent gradual quenching upon lowering pH from 8.0 to 5.5. The order of pH dependent efficiency of quenching for tC probes was 6tC < 3tC < 1tC. This efficiently quenched fluorescence of tC probes was ascribed to the protonated tC-H^+^ fluorophore that was generated upon i-motif formation. Temperature dependence of tC probe fluorescence exhibited profiles that were dependent on the pH value showing interplay of two competing processes, dequenching associated with unfolding and temperature induced nonradiative deactivation of excited state. Analytical usability of the new pH-sensitive aptamer probes were evidenced by monitoring reversible changes of fluorescence upon cyclic changes in pH between values 7.5 and 5.5. Further studies concerning optimizing of tC probes, also by multiple labeling of i-motif forming aptamers with fluorescent analogs of cytosine as well as attempts to monitor pH changes in biological objects are in progress.
